# Efficient degradation of neomycin by *Bacillus velezensis* and *Cupriavidus basilensis* isolated from mangrove soil and pharmaceutical wastewater

**DOI:** 10.3389/fmicb.2025.1544888

**Published:** 2025-01-29

**Authors:** Qian Yang, Wenzhuan Huang, Xue Yan, Qiang Ding, Jiaxin Liu, Bo Cheng, Tao Duan

**Affiliations:** ^1^School of Environmental Ecology and Biological Engineering, Wuhan Institute of Technology, Wuhan, China; ^2^Yichang Humanwell Pharmaceutical Co., Ltd., Hubei, China; ^3^Guangzhou Higher Education Mega Center, School of Life Science and Biopharmaceutics, Guangdong Pharmaceutical University, Guangzhou, China

**Keywords:** *Bacillus velezensis*, *Cupriavidus basilensis*, neomycin-free soil, neomycin, biodegradation

## Abstract

Neomycin, an aminoglycoside antibiotic, is widely utilized for veterinary medicine in disease prevention. Biodegradation is a key pathway for the removal of neomycin from the environment. To date, only the white-rot fungus *Trametes versicolor* and the ericoid mycorrhizal fungus *Rhizoscyphus ericae* have been documented to efficiently degrade neomycin. However, no bacterial species with neomycin-degrading capabilities have been reported, underscoring a significant gap in microbial research related to neomycin remediation. In this study, *Cupriavidus basilensis* and *Bacillus velezensis* were isolated from pharmaceutical wastewater and neomycin-free mangrove soil through enrichment culture and gradual acclimatization, respectively. These isolates demonstrated neomycin degradation rates of 46.4 and 37.6% in 96 h with 100 mg·L^−1^ neomycin as the sole carbon source. *Cupriavidus basilensis* achieved a degradation rate of 50.83% with ammonium sulfate supplementation, while *Bacillus velezensis* exhibited a superior degradation efficiency of 58.44% with soluble starch. Our findings offer valuable insights into the microbial degradation of neomycin. Two neomycin-degrading bacteria were isolated for the first time. Both species degraded neomycin as the sole carbon source or under co-metabolic conditions within 4 days. Microorganisms from neomycin-free environments adapted to neomycin stress and outperformed those from contaminated sources. This challenges the assumption that antibiotic-degrading microorganisms mainly originate from polluted environments. The findings expand the diversity of known neomycin-degrading microorganisms and demonstrate their potential for removing refractory neomycin from pharmaceutical wastewater.

## 1 Introduction

Antibiotics are widely utilized as antimicrobial agents in both human healthcare and veterinary medicine (Yang et al., 2021). Global antibiotic consumption increased by 16.3% from 2016 to 2023, with projections indicating a potential 52.3% increase by 2030 (Klein et al., 2024). The misuse and overuse of antibiotics have fueled the emergence of multidrug-resistant pathogens, posing significant threats to public and animal health (Patangia et al., 2022). Antibiotic pollution primarily originates from pharmaceutical wastewater, aquaculture, livestock farming, and landfill leachate (Anh et al., 2021). Neomycin (Neo), an aminoglycoside antibiotic, is extensively used in livestock and poultry production (Low et al., 2021). The expansion of farming practices has further escalated the demand for veterinary antibiotics (Guo et al., 2021; Shao et al., 2021; Li S. et al., 2024). Besides agricultural runoff, pharmaceutical manufacturing is a major contributor to aminoglycoside antibiotic pollution (Tang et al., 2020). The extraction processes of antibiotics release substantial amounts of contaminated wastewater into natural ecosystems, exacerbating antibiotic resistance and promoting the spread of resistance genes (Han et al., 2024). Antibiotic concentrations in pharmaceutical effluents can reach mg·L^−1^ levels (Sim et al., 2011; Li K. et al., 2024). In response, the World Health Organization (WHO) issued its first guidelines on pharmaceutical antibiotic pollution, advocating preventive measures across municipal systems, manufacturing, healthcare, and agri-food sectors (WHO, 2024).

The thermal stability and resistance to acidic and alkaline conditions of aminoglycoside antibiotics render conventional removal methods ineffective (Nian et al., 2023). In contrast, microorganisms produce specific enzymes such as aminoglycoside acetyltransferases, phosphotransferases, and nucleotidyltransferases that deactivate aminoglycosides, thereby enabling microbial survival in environments with high antibiotic concentrations (Li et al., 2012; Liu et al., 2017; Cox et al., 2018; Stenholm et al., 2022). Moreover, microorganisms capable of mineralizing aminoglycosides have been identified in various ecosystems (Zeng et al., 2019), which has spurred interest in the microbial degradation of these compounds (Apreja et al., 2022). Most research has focused on the degradation of gentamicin and kanamycin. For example, *Stenotrophomonas maltophilia* and *Pseudomonas* sp. have been shown to degrade streptomycin (Fenton et al., 1973; Demars et al., 2024); *AMQD4* bacterial consortia can degrade gentamicin (Liu et al., 2017); and *Aquamicrobium* sp. I-A can degrade kanamycin (Chen et al., 2023). These microorganisms are typically isolated from antibiotic-contaminated soils, water sources, or pharmaceutical wastewater. The feasibility of isolating Neo-degrading microorganisms has also been proposed. However, Neo presents unique challenges due to its stable aminocyclitol ring, which confers strong resistance to microbial enzymatic cleavage (Obszynski et al., 2022). Only *Basidiomycetes* have been reported to biodegrade Neo (Stenholm et al., 2022), but their long growth cycle (up to 12 days) results in low degradation efficiency.

It is widely accepted that microorganisms exposed to antibiotic pollution are more likely to develop antibiotic-degrading capabilities. However, recent studies have identified antibiotic-degrading microorganisms in environments devoid of antibiotic pollution, challenging this conventional view. For instance, *E. coli* and *Cellulomonas* sp., isolated from antibiotic-free soils, were found to survive using aminoglycoside antibiotics as their sole carbon source (Dantas et al., 2008; Bello González et al., 2016). Similarly, *Stenotrophomonas maltophilia* isolated from antibiotic-free soils demonstrated efficient degradation of streptomycin (Bello González et al., 2016; Reis et al., 2020). While horizontal gene transfer (HGT) of resistance genes has been proposed as a mechanism for this phenomenon (Khmelevtsova et al., 2020; Liu et al., 2022), evidence suggests that antibiotic degradation may not always depend on HGT. In a study by Zhang et al., a strain capable of utilizing Neo as its sole carbon source was isolated from soil without prior exposure to Neo. Although aph3’ and aac (3)-Ia aminoglycoside resistance genes were detected in the soil, these genes were absent in the isolated strain itself (Zhang and Dick, 2014). This finding indicates that microbial antibiotic degradation capabilities can exist independently of HGT mechanisms. Consequently, it highlights the potential to isolate highly efficient antibiotic-degrading microorganisms from microbial reservoirs such as soil that have not been exposed to antibiotics. Based on this, we aimed to isolate Neo-degrading bacteria from both pharmaceutical wastewater with long-term exposure to high concentrations of Neo and from soil that had never been exposed to Neo, to evaluate their biodegradation capabilities and degradation characteristics.

## 2 Materials and methods

### 2.1 Chemicals and medium

Neo (purity ≥ 97%) used in this study was procured from Yichang Sanxia Pharmaceutical Co., Ltd. Glucose was sourced from Tianjin Kaitong Chemical Reagent Co., Ltd. Soluble starch, ammonium citrate, ammonium sulfate, and defatted soy flour were obtained from China National Pharmaceutical (Group) Shanghai Chemical Reagent Company. Peptone was supplied by Biosharp. The Luria-Bertani (LB) medium, utilized for enrichment, comprised 5 g·L^−1^ NaCl, 10 g·L^−1^ yeast extract, and 10 g·L^−1^ peptone, adjusted to a pH of 7.2. Domestication and degradation experiments were conducted using a modified M9 medium, which lacked carbon and nitrogen sources, to screen for Neo-efficient degrading strains. The composition of the modified M9 medium included 0.45 g·L^−1^ KH_2_PO_4_, 0.1 g·L^−1^ MgSO_4_·7H_2_O, and 1.79 g·L^−1^ K_2_HPO_4_. To prepare LB solid medium, 2.0% (w/v) agar powder was added to the LB medium. All media were sterilized prior to use.

### 2.2 Determination of Neo concentration

The Neo concentration in soil and wastewater samples was analyzed using a Thermo Scientific Dionex U3000 High-Performance Liquid Chromatography (HPLC) system equipped with an ELSD 6000 Evaporative Light Scattering Detector. Separation was achieved on an Apollo C18 column (4.6 mm × 250 mm, 5 μm particle size) at a column temperature of 25°C. The mobile phase consisted of a mixture of 2.0% trifluoroacetic acid and 0.1% heptafluorobutyric acid (20:1, v/v), filtered through a 0.22 μm membrane. Each sample injection volume was set to 20 μL, and the flow rate was maintained at 0.6 mL·min^−1^. The evaporative light-scattering detector was configured with an evaporation temperature of 110°C and a gas flow rate of 3.0 mL·min^−1^ The gain value was set to 1 DT, and the impaction mode was turned off. The total run time for each analysis was 20 min.

**Figure 1:**
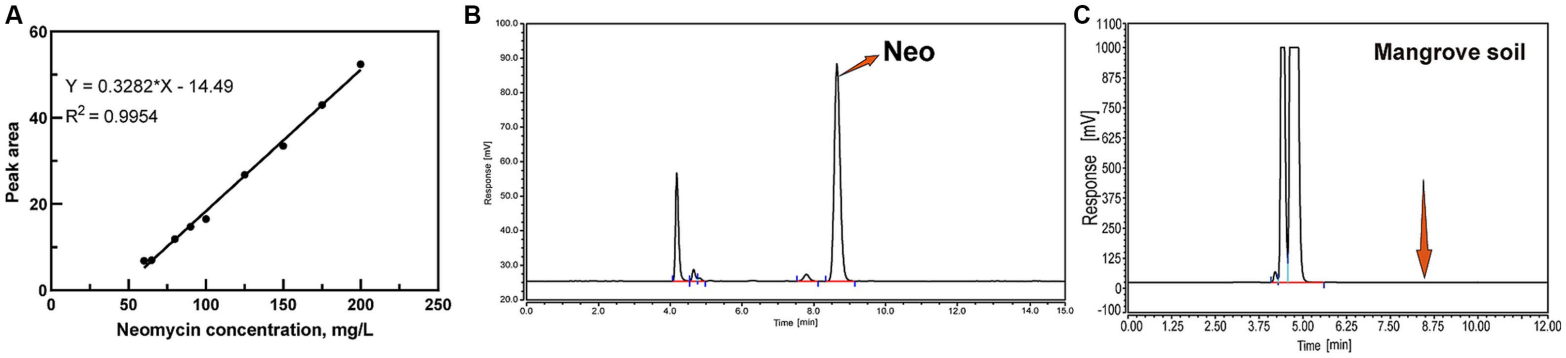
HPLC analysis of Neo. **(A)** Standard curve for Neo; **(B)** Chromatogram of Neo; **(C)** Neo content in Mangrove soil.

For liquid samples, Neo extraction involved centrifuging at 9,040× g for 10 min. The supernatant was then mixed with Na_2_EDTA-McIlvaine buffer to chelate metal ions (He et al., 2021). For soil samples, 20 mL of extraction solution (methanol: water = 1:1, v/v) was added to sieved soil, followed by ultrasonication for 30 min and centrifugation at 9040× g for 10 min. The resulting supernatants were filtered through a 0.22 μm membrane and stored at −20°C for further analysis (Yu et al., 2019; He et al., 2021). The linear regression equation for Neo standard solutions was determined as *y* = 0.3282*x* – 14.49 (Figure 1A), where *y* represents peak area and *x* represents Neo concentration. The chromatographic profile of Neo exhibited consistent retention times at 8.645 ± 0.05 min (Figure 1B).

The degradation rate was calculated as follows:


$$\mathrm{Degradation rate}=\frac{\begin{array}{l} \mathrm{initial}\;Neo\;\mathrm{concentration}-\\ \mathrm{residual}\;Neo\;\mathrm{concentration} \end{array}}{\mathrm{initial}\;Neo\;\mathrm{concentration}}\times 100\%$$

### 2.3 Sample sources and preparation

Shenzhen Longgang District (N22.48894, E114.58263), situated within the city of Shenzhen, is a critical region for mangrove distribution. The Shenzhen Bay Mangrove Wetland serves as an essential ecological conservation area. Previous studies have indicated that many mangrove wetlands in Shenzhen face significant ecological risks due to contamination from various pollutants (Lingyun et al., 2019). Soil samples were collected from three distinct locations, excluding the topsoil layer and sampling at depths between 5 and 20 cm. The collected soil samples were air-dried for 7 days, subsequently ground into a fine powder, and stored in sterile sealed bags at temperatures below −20°C until further analysis. HPLC analysis revealed no detectable Neo residues in the soil samples (Figure 1C). Wastewater samples were obtained from a pharmaceutical factory in Hubei Province, China, which manufactures Neo through a fermentation-based process. Biochemical hydrolysis effectively eliminates microorganisms that cannot tolerate antibiotics, rendering the treated wastewater suitable for this study.

### 2.4 Enrichment culture of neo-tolerant microorganisms

The LB medium supplemented with 100 mg·L^−1^ Neo was used to enrich the isolates. This concentration of Neo mimics the high levels typically encountered in pharmaceutical wastewater treatment processes. Initially, 1 mL of pharmaceutical wastewater or 1 g of soil was suspended in 9 mL of sterile distilled water and mixed thoroughly for 1 min. The suspension was then serially diluted 10^6^-fold using sterile distilled water. Subsequently, a 10% (v/v) inoculum of the diluted suspension was added to 100 mL of sterile LB medium containing 100 mg·L^−1^ Neo. Cultures were incubated at 35°C with shaking at 220 rpm for 7 days until an optical density (OD_600_) value of 1.000 was achieved. These cultures served as the inoculum for subsequent domestication steps.

### 2.5 Domestication of neo-tolerant microorganisms

To acquire strains capable of utilizing Neo as the sole carbon source, 10 mL of the enriched bacterial suspension was inoculated into 90 mL of optimized M9 medium supplemented with 50 mg·L^−1^ Neo. Cultures were incubated at 35°C with shaking at 220 rpm for 7 days to complete the first domestication cycle. For subsequent cycles, 10 mL of the previous culture was transferred to 90 mL of fresh optimized M9 medium, with Neo concentrations incrementally increased by 50 mg·L^−1^ per cycle, reaching 200 mg·L^−1^ by the fourth cycle. OD_600_ values was measured following each acclimation period.

### 2.6 Isolation, purification, and 16S rRNA identification of neo-degrading strains

The final domesticated culture was serially diluted 10^3^-fold with sterile distilled water and plated onto LB agar plates. The cultures were incubated at 35°C for 48 h. Distinct colonies based on morphology were isolated using the streak plate method for further purification. This purification process was repeated three times to ensure pure cultures. The purified strains were then inoculated onto fresh LB agar plates and incubated at 35°C for 18 h. Crystal violet staining and bacterial morphology were examined under a biological microscope (MODEL-ECLIPSE-E200, Nikon Corporation, Shanghai, China). Genomic DNA was extracted from the purified strains using the MGIEasy Bacterial DNA Extraction Kit (MD01T-96, MGI, Wuhan, China). The 16S rRNA gene was amplified using universal primers 27F and 1492R (Yu et al., 2013). PCR amplification conditions included an initial denaturation at 96°C for 5 min, followed by 35 cycles of denaturation at 96°C for 30 s, annealing at 56°C for 30 s, and extension at 72°C for 1 min, with a final extension at 72°C for 10 min. PCR products were verified by agarose gel electrophoresis and subsequently sent to BGI (Beijing Genomics Institute) for sequencing. The obtained sequences were analyzed using the BLAST tool (https://www.ncbi.nlm.nih.gov/) for species identification. A phylogenetic tree was constructed using the Neighbor-Joining method in MEGA 11.0 software. The nucleotide sequences have been deposited in the GenBank database (SH1: SUB14883397 and RS2: SUB14885154).

### 2.7 Neo biodegradation ability of strains SH1 and RS2

The purified cultures of strain SH1 and strain RS2 were grown in LB medium until they reached the logarithmic growth phase. Cells were harvested and washed three times with 0.9% NaCl solution. The OD_600_ values of the cell suspensions was adjusted to 1.000 by diluting with fresh LB medium, and measurements were conducted using a SpectraMaxiD3 Microplate Reader (Shanghai Minggujia Electronic Technology Co., Ltd.). Subsequently, the bacterial suspensions were inoculated at a 10% inoculum size into modified M9 medium containing 100 mg·L^−1^ Neo, with an initial pH of 7.2, for biodegradation assays (Yin et al., 2020). This Neo concentration was selected based on preliminary studies, as concentrations exceeding 100 mg·L^−1^ significantly inhibited bacterial growth during strain domestication (Supplementary Table S1). Samples were collected every 24 h to monitor Neo residuals and OD_600_ values (Li et al., 2021). A control group with the same Neo concentration in M9 medium was established to minimize potential external influences.

### 2.8 Study of Neo biodegradation characteristics

To optimize the cultivation conditions for the Neo-degrading strains SH1 and RS2, we systematically evaluated the effects of varying substrate concentrations (100, 500, and 1,000 mg·L^−1^), nitrogen sources (ammonium sulfate, peptone, and defatted soy flour), and carbon sources (citric acid ammonium, glucose, and soluble starch) on degradation efficiency. Each condition was tested in triplicate with a blank control group included. Bacterial suspensions were collected at multiple time points over a 96 h incubation period, and OD_600_ values were measured to assess bacterial growth. After sample processing, Neo concentrations were quantified using HPLC, and degradation rates were subsequently calculated.

#### 2.8.1 Effect of substrate concentration on growth and degradation rates of strains SH1 and RS2

Neo solutions with initial concentrations of 100, 500, and 1,000 mg·L^−1^ were added to M9 medium adjusted to pH 7.2. A 10% (v/v) inoculum of the bacterial suspension was introduced into the medium, and the cultures were incubated at 35°C for 96 h. Uninoculated samples served as controls. Each condition was tested in triplicate. After the 96 h incubation period, OD_600_ values and residual Neo concentrations were measured.

#### 2.8.2 Effect of nitrogen sources on growth and degradation rates of strains SH1 and RS2

Neo solutions at a concentration of 100 mg·L^−1^ were added to the M9 medium, which was adjusted to pH 7.2. Ammonium sulfate, peptone, and defatted soy flour were incorporated into the medium at concentrations of 0.1 g·L^−1^ each. A 10% (v/v) inoculum of the bacterial suspension was introduced into the medium, and the cultures were incubated at 35°C for 96 h. Uninoculated samples served as controls. Each condition was tested in triplicate. Following the 96 h incubation period, OD_600_ values and residual Neo concentrations were measured.

#### 2.8.3 Effect of carbon sources on growth and degradation rates of strains SH1 and RS2

A 100 mg·L^−1^ Neo solution was added to the M9 medium adjusted to pH 7.2. Ammonium citrate, glucose, and soluble starch were each added at a concentration of 0.1 g·L^−1^. A 10% (v/v) inoculum of the bacterial suspension was introduced into the cultures, which were then incubated at 35°C for 96 h. Uninoculated samples served as controls. Each condition was tested in triplicate. After the 96 h incubation period, OD_600_ values and residual Neo concentrations were measured to evaluate the impact of different carbon sources on bacterial growth and Neo degradation efficiency.

## 3 Results and discussion

### 3.1 Isolation, purification, and 16S rRNA identification of neo-degrading strains

**Figure 2:**
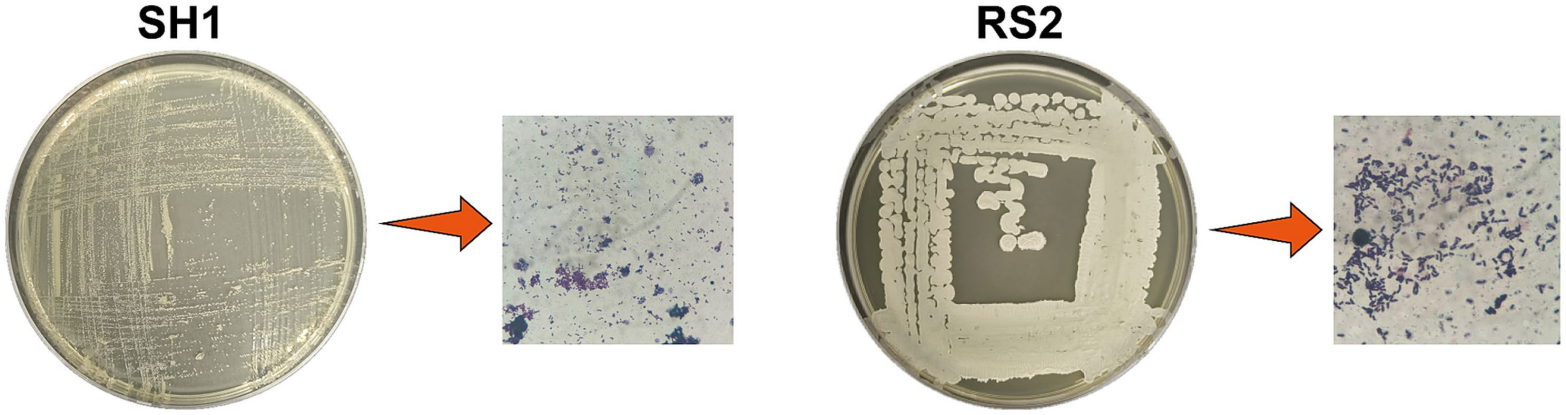
Morphology and Gram staining of screening strains.

Through enrichment culture and gradual domestication using Neo-contaminated pharmaceutical wastewater and Neo-free mangrove soil samples, two strains capable of utilizing Neo as the sole carbon source were isolated. These strains were designated as SH1 and RS2. The strains were purified, stained with crystal violet, and characterized based on their morphological features. Strain SH1 was identified as a Gram-negative short rod with small, smooth, raised colonies, while strain RS2 was identified as a Gram-positive rod with larger, wrinkled, raised colonies (Figure 2).

**Figure 3:**
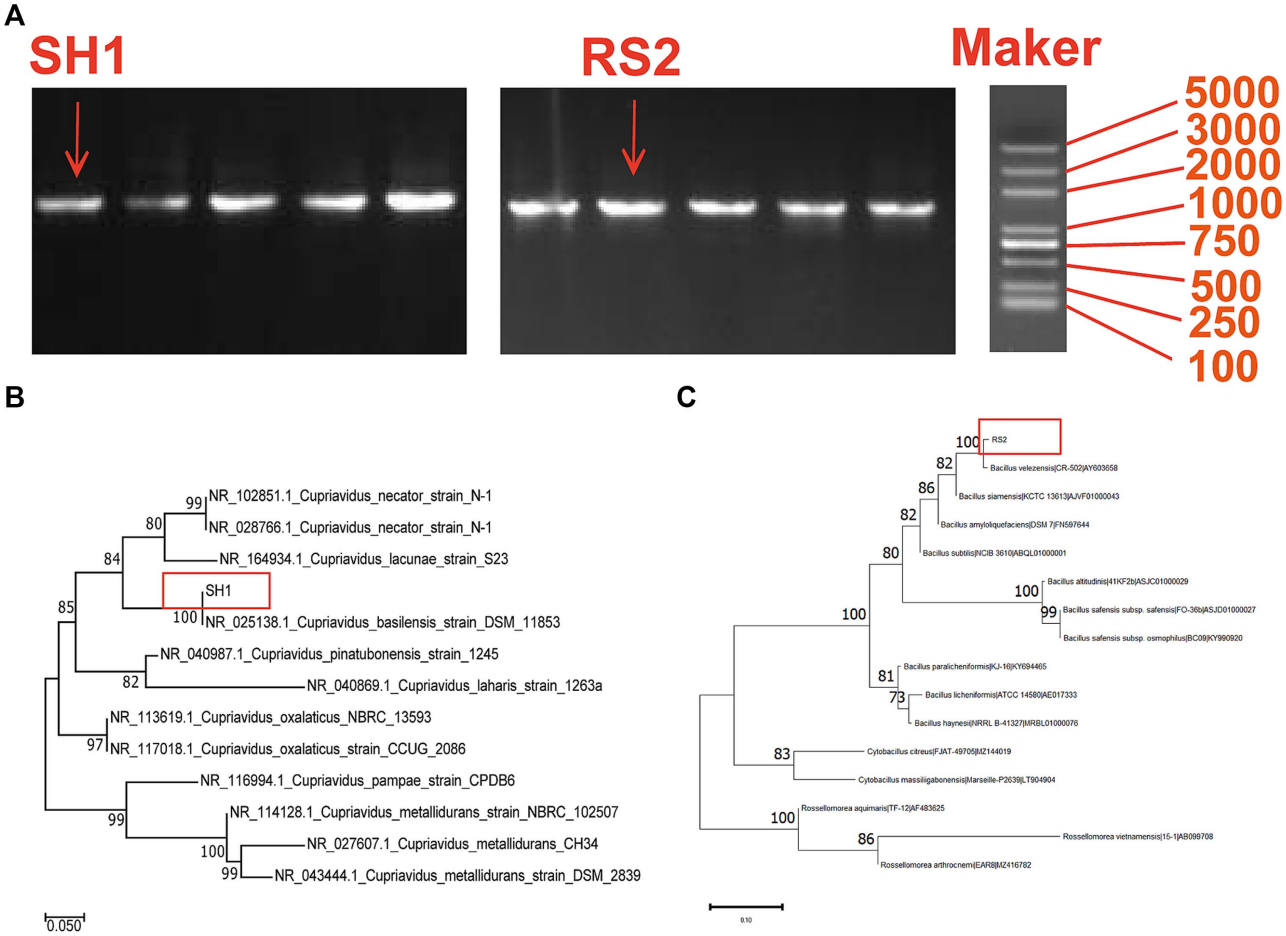
Molecular biological analysis of strain SH1 and RS2: **(A)** PCR electrophoresis product of strain SH1 and RS2; **(B)** Phylogenetic tree for the strain SH1 and related bacterial strains; **(C)** Phylogenetic tree for the strain RS2 and related bacterial strains.

Strains SH1 and RS2 were identified as *Cupriavidus basilensis* and *Bacillus velezensis*, respectively (Table 1). Molecular biological analysis revealed that PCR-amplified DNA fragments from strains SH1 and RS2 exhibited consistent single-band patterns, with lengths of approximately 1,360 bp and 1,410 bp, respectively (Figure 3). Sequencing confirmed full-length gene sequences of 1,368 bp and 1,413 bp for strain SH1 and strain RS2, respectively, corroborating the electrophoresis results (Figure 3A). BLAST comparisons of the 16S rRNA sequencing results showed 100% sequence similarity for both strains. Based on these findings, a phylogenetic tree was constructed (Figures 3B,C).

**Table 1:** Homology comparison of 16S rRNA gene sequences of screened strains.

Strains number	The closest species		Identity %	Genus submission
	Latin name	Accession No.		
SH1	*Cupriavidus basilensis*	CP062804.1	100	SUB14883397
RS2	*Bacillus velezensis*	CP054714.1	100	SUB14885154

### 3.2 Neo biodegradation ability of strains SH1 and RS2

**Figure 4:**
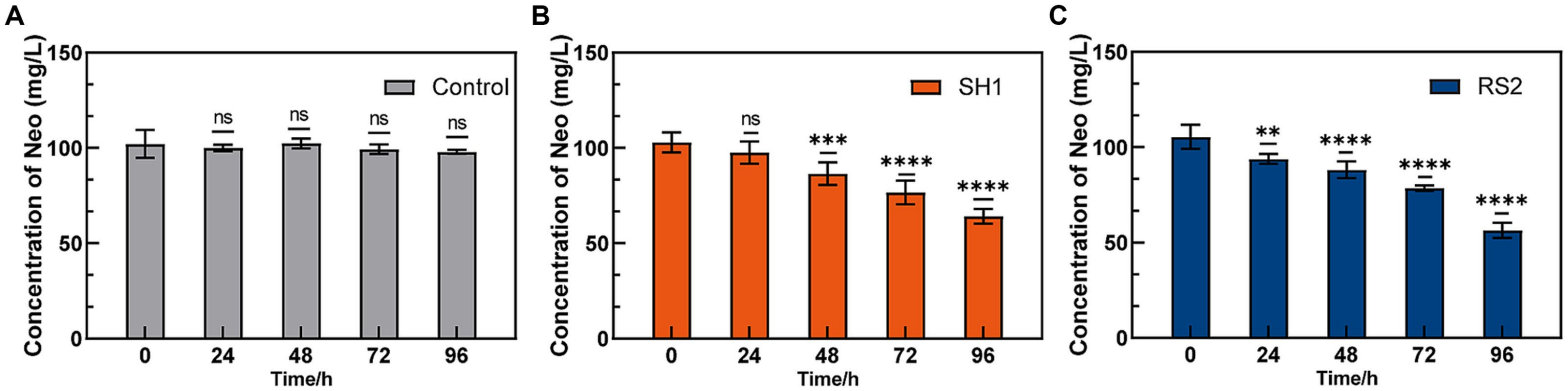
Neo degradation ability of enriched strains. **(A)** Control; **(B)** SH1; **(C)** RS2.

Strains SH1 and RS2 demonstrated 96-h Neo degradation rates of 37.67 and 46.49%, respectively, when Neo was used as the sole carbon source at a concentration of 100 mg·L^−1^ (Figure 4). Previous studies have shown that several *Cupriavidus* species can effectively degrade ochratoxin A (Ferenczi et al., 2014), bisphenol A (Fischer et al., 2010), and assist in copper bioremediation (Kugler et al., 2022). Similarly, some *Bacillus* species have been identified as capable of degrading sulfamethoxazole (Lin et al., 2024) and tetracycline (Al-Dhabi et al., 2021). This study is the first to report that *Cupriavidus basilensis* and *Bacillus velezensis* can degrade Neo. Most antibiotic-degrading strains are isolated from antibiotic-contaminated environments such as wastewater, solid waste, and soils (Table 2). Wang et al. isolated sulfadiazine-degrading bacteria from antibiotic-contaminated soils and found that sulfadiazine resistance genes played a key role in the degradation process (Wang et al., 2024). Additionally, Rodríguez-Verdugo et al. evolved *Escherichia coli* B under heat stress (42.2°C) over 2000 generations in an environment free of rifampicin (Rodríguez-Verdugo et al., 2013), resulting in spontaneous mutations in the *rpoB* gene, which conferred rifampicin resistance. This suggests that gene mutations leading to antibiotic resistance can occur even in environments without antibiotics, depending on the mutation background and environmental conditions. In this study, we have discovered and isolated neo-degrading bacteria from environments free of Neo. Specifically, *Bacillus velezensis* was isolated from Neo-free soil, while *Cupriavidus basilensis* was isolated from Neo-pharmaceutical wastewater, both exhibiting Neo degradation abilities. The degradation ability of these strains may be linked to Neo resistance genes.

**Table 2:** Antibiotics from different sources degrade microorganisms.

Antibiotic	Microorganisms	Sample source	References
Streptomycin	*Stenotrophomonas maltophilia*	Soil-dwelling	Fenton et al. (1973)
	*Pseudomonad bacterium*	Estrogen-containing urban soil	Demars et al. (2024)
Gentamicin	*AMQD4*	Gentamicin-containing sludge	Liu et al. (2017)
	*Aspergillus terreus* FZC3	Gentamicin produces solid waste and wastewater	Liu et al. (2016)
Kanamycin	*Domestication strain* DSM, *Aquamicrobium* sp. I-A	Antibiotic contaminated soil and Wastewater	Chen et al. (2023)
Tetracycline	*Arthrobacter nicotianae* OTC-16	Activated sludge from pharmaceutical Wastewater treatment plants	Shi et al. (2021)
	*Pseudomonas* sp. DX-21	Sludge of a long-operating SBR	Yang et al. (2024)
Erythromycin	*Paracoccus versutus* W7	Sewage sludge for municipal sewage treatment	Ren et al. (2023)
Chloramphenicol	*Klebsiella* sp. YB1	Earthworm gut content	Tan et al. (2023)
Sulfamethoxazole	*Proteus mirabilis* sp. ZXY4	Sludge from sewage treatment plants	Yan et al. (2023)

### 3.3 Effects of different culture conditions on Neo biodegradation by SH1 and RS2

**Figure 5:**
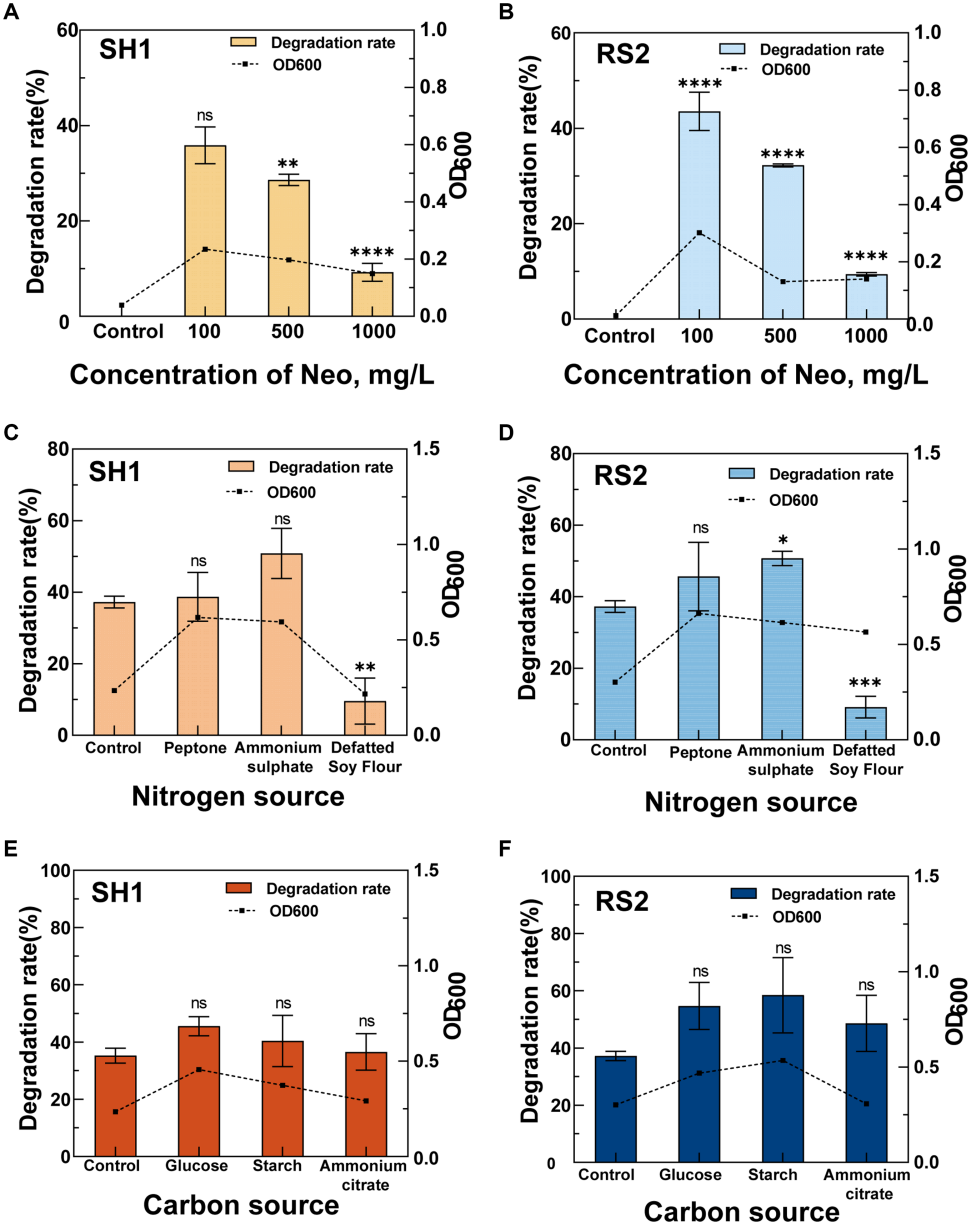
Effects of different conditions on the Neo degradation rate of strains SH1 and RS2. **(A,B)** Concentration of Neo; **(C,D)** Nitrogen source; **(E,F)** Carbon source.

When Neo served as the sole carbon source, strains SH1 and RS2 exhibited differential degradation efficiencies over 96 h at concentrations ranging from 100 to 1,000 mg·L^−1^ (Figures 5A,B). As the Neo concentration increased, both the degradation rate and bacterial growth were progressively inhibited. Specifically, at an initial Neo concentration of 100 mg·L^−1^, optimal degradation rates and growth conditions were observed for strains SH1 and RS2, with degradation rates reaching 35.83 and 43.54%, respectively, and OD_600_ values attaining 0.235 and 0.302. These findings suggest that the initial substrate concentration significantly affects the efficiency of Neo biodegradation. The high degradation rate at 100 mg·L^−1^ can likely be attributed to enhanced bacterial growth and the secretion of functional enzymes involved in biotransformation (Sánchez-San Martín et al., 2024). Conversely, at higher initial concentrations of 500 mg·L^−1^ and 1,000 mg·L^−1^, metabolic overload may have occurred, resulting in the accumulation of metabolites or by-products that inhibited bacterial growth and reduced degradation efficiency (Chen et al., 2022).

In the optimized M9 medium containing 100 mg·L^−1^ Neo, we investigated the effects of different nitrogen sources—ammonium sulfate, peptone, and defatted soy flour—on Neo degradation by strains SH1 and RS2 (Figures 5C,D). Compared to the control, defatted soy flour significantly inhibited Neo degradation in both strains, resulting in degradation rates of only 9.54% for SH1 and 9.14% for RS2. The underlying mechanism of this inhibition warrants further investigation. In contrast, ammonium sulfate enhanced the Neo degradation ability of both strains, achieving the highest degradation rates of 50.83% for SH1 and 50.71% for RS2. When bacteria utilize inorganic nitrogen sources such as ammonium sulfate for metabolism, they require additional carbon sources (Hargreaves, 2006), which may explain the observed increase in Neo degradation rates. This finding is consistent with previous studies on sulfonamide biodegradation, where sodium nitrate and ammonium chloride were shown to promote biodegradation (Vijayaraghavan et al., 2021). Unlike glucose, Neo served as the sole carbon source in our study, suggesting that inorganic nitrogen might enhance carbon metabolism similarly.

The effects of different carbon sources—ammonium citrate, glucose, and soluble starch—on the Neo degradation ability of the two strains at a 100 mg·L^−1^ Neo concentration (Figures 5E,F) were investigated. Compared to the control group (without additional carbon sources), all three carbon sources significantly increased the degradation rates to varying extents, indicating that these carbon sources can enhance Neo removal by the bacteria (Leng et al., 2016; He et al., 2021). Specifically, strain SH1 exhibited the highest Neo degradation rate of 45.53% when supplemented with glucose. Strain RS2 also showed enhanced growth and Neo degradation ability in the presence of glucose and soluble starch, achieving degradation rates of 54.69 and 58.44%, respectively. The addition of appropriate co-substrates can activate functional enzymes (Zhang et al., 2020) and accelerate the biodegradation of recalcitrant organic compounds (Barnhill et al., 2010).

## 4 Conclusion

In this study, *Cupriavidus basilensis* and *Bacillus velezensis* were isolated from Neo pharmaceutical wastewater and from soil that had not been exposed to Neo. These isolates demonstrated the ability to utilize Neo as the sole carbon source. The degradation experiments revealed that both *Cupriavidus basilensis* and *Bacillus velezensis* exhibited significant Neo degradation capabilities. Specifically, *Cupriavidus basilensis* achieved a maximum degradation rate of 50.83% for 100 mg·L^−1^ Neo within 96 h when ammonium sulfate was used as the nitrogen source. Meanwhile, *Bacillus velezensis* reached a maximum degradation rate of 58.43% under the same Neo concentration when soluble starch served as the additional carbon source.

The results demonstrate that substrate concentration significantly influences the efficiency of Neo degradation, with appropriate co-substrates promoting bacterial growth and enhancing Neo biodegradation. The identification of *Cupriavidus basilensis* and *Bacillus velezensis* as Neo-degrading strains expands the known range of microorganisms capable of degrading Neo. These findings suggest their potential utility in the bioremediation of Neo-contaminated soils and wastewater. However, further studies are required to evaluate their performance under actual environmental conditions.

## Data Availability

The datasets presented in this study can be found in online repositories. The names of the repository/repositories and accession number(s) can be found in the article/Supplementary material.
