# Supplementary material for: Efficient degradation of neomycin by Bacillus velezensis and Cupriavidus basilensis isolated from mangrove soil and pharmaceutical wastewater
Source: Front Microbiol. 2025 Jan 29;16:1544888. doi: 10.3389/fmicb.2025.1544888 (PMC11817266; doi:10.3389/fmicb.2025.1544888)
Supplement: Supplementary file 1 [file Table_1.docx]

Supplementary Material

# Supplementary Tables

**Supplementary Table 1.** The growth of the strain SH1 and strain RS2 after 7 days of domestication with different concentrations of Neo.

| Concentrations of Neo | 50 mg·L^-1^ | 100 mg·L^-1^ | 150 mg·L^-1^ | 200 mg·L^-1^ |
| --- | --- | --- | --- | --- |
| Strain SH1 | 0.296 ± 0.020 | 0.520 ± 0.024 | 0.137 ± 0.016 | 0.086 ± 0.009 |
| Strain RS2 | 0.385 ± 0.019 | 0.617 ± 0.031 | 0.106 ± 0.016 | 0.069 ± 0.004 |
